# Correlation Between Breath Acetone and Ketone Bodies in Blood and Urine Among Individuals with Different Glycometabolic Statuses Based on PTR-TOF-MS

**DOI:** 10.3390/diagnostics16132043

**Published:** 2026-06-30

**Authors:** Ting Li, Yanting Yang, Chen Huang, Hanlu Yue, Li Chen, Yan Huang, Fengming Luo

**Affiliations:** 1 Health Management Center, General Practice Medical Center, West China Hospital, Sichuan University, Chengdu 610041, China; 2 Aliben Sci & Technol Co., Ltd., Chengdu 611930, China; 3 Department of Biotherapy, Cancer Center and State Key Laboratory of Biotherapy, West China Hospital, Sichuan University, Chengdu 610041, China; 4 State Key Laboratory of Respiratory Health and Multimorbidity, West China Hospital, Sichuan University, Chengdu 610041, China; 5 Research Laboratory for Prediction and Evaluation of Chronic Diseases in the Elderly, National Clinical Research Center for Geriatric Diseases, West China Hospital, Sichuan University, Chengdu 610041, China; 6 General Practice Research Institute, West China Hospital, Sichuan University, Chengdu 610041, China; 7 Department of Pulmonary and Critical Care Medicine, West China Hospital, Sichuan University, Chengdu 610041, China; 8 Department of Respiratory and Critical Care Medicine, Laboratory of Pulmonary Immunology and Inflammation, Frontiers Science Center for Disease-Related Molecular Network, West China Hospital, Sichuan University, Chengdu 610041, China

**Keywords:** ketone bodies, breath acetone, β-hydroxybutyrate, proton transfer reaction coupled to time-of-flight mass spectrometry (PTR-TOF-MS), different glycometabolic status

## Abstract

**Background/Objectives**: Rapid and accurate ketone detection is clinically valuable for both diabetic and non-diabetic individuals. Breath acetone represents an attractive non-invasive and real-time alternative for monitoring ketones. This study aimed to explore the correlation between breath acetone measured by proton transfer reaction time-of-flight mass spectrometry (PTR-TOF-MS) and ketone bodies in blood and urine in individuals with normoglycemia, prediabetes and diabetes. **Methods**: A total of 4000 participants were recruited. Urinary ketone levels, blood β-hydroxybutyrate (βHB) concentrations, and breath acetone (measured by PTR-TOF-MS) were assessed. Participants were classified into urine ketone-negative and urine ketone-positive groups. They were further divided according to the semiquantitative degree of urinary ketone (−, +, ++, +++ and ++++). Spearman rank correlation analysis was performed to evaluate the relationship between breath acetone and blood βHB. Receiver operating characteristic (ROC) curve analysis was conducted to determine the preliminary thresholds of breath acetone for detecting blood βHB positivity. Multiple linear regression, including a quadratic term for blood βHB, was performed, and the inflection point of the curve was calculated. **Results**: Blood βHB and breath acetone were significantly higher in the urine ketone-positive group than in the urine ketone-negative group (*p* < 0.001). A progressive elevation in blood βHB and exhaled breath acetone concentrations was observed with increasing urinary ketone levels (*p* < 0.001). The Spearman rank correlation coefficient between breath acetone and blood βHB was 0.493, 0.522, 0.379, and 0.636 in the general population, normoglycemia, prediabetes, and diabetes groups, respectively (all *p* < 0.001). The preliminary thresholds of breath acetone concentration for detecting blood βHB positivity were 2.37 ppmv in diabetes, 1.10 ppmv in prediabetes, and 2.05 ppmv in normoglycemia, respectively. Blood βHB exhibited a significant quadratic association with breath acetone in the general population, normoglycemia, and diabetes (B = −1.698, −1.409, and −16.070, respectively; *p* < 0.001), with inflection points of the quadratic curves at 2.73 mmol/L, 3.28 mmol/L and 1.61 mmol/L, respectively, whereas in prediabetes, the relationship did not significantly deviate from linearity. **Conclusions**: In normoglycemia and diabetes, breath acetone based on PTR-TOF-MS exhibited an inverted U-shaped relationship with blood βHB, while in prediabetes, breath acetone showed a linear positive correlation with blood βHB. In populations where blood βHB is below the quadratic inflection point, breath acetone measured by PTR-TOF-MS holds promise as a non-invasive ketone-monitoring tool.

## 1. Introduction

Ketone bodies, as products of the fat metabolism process, include three molecules, acetoacetate (AcAc), β-hydroxybutyrate (βHB) and acetone [1,2]. The liver is the site of ketone body formation, and the first ketone body produced is AcAc, which is reduced to βHB or acetone. Studies have shown that ketone bodies not only serve as liver-derived alternative fuel sources during periods of carbohydrate restriction but also act as toxic mediators during diabetic ketosis and modulate cellular homeostasis in multiple physiological states [3]. Marked elevations of ketone bodies are seen in certain physiological and pathophysiological states, such as pregnancy, ketogenic diets, prolonged starvation and exercise, severe insulin resistance with impaired glucose utilization, and diabetic ketoacidosis (DKA) [1,4]. Meanwhile, increased ketone body metabolism is a feature of heart failure, and ketone body production may reduce inflammation and delay the progression of several kidney diseases [5,6]. Changes in ketone body concentration directly reflect energy metabolism status and are closely related to the functions of the heart and kidneys. Therefore, rapid and accurate detection of ketone bodies holds significant clinical value not only for patients at risk of diabetic ketoacidosis but also for non-diabetic individuals.

Currently, the most widely used clinical methods for ketone body detection include blood βHB measurement and urine ketone strip testing. However, blood testing requires invasive sampling and assay time, making it impractical for repeated measurements at short intervals [7]. Urine testing, which provides semi-quantitative results, exhibits a time lag, reflecting metabolic status from hours earlier and is inaccurate in patients with renal dysfunction [2]. Normal and pathological status of physiological processes in the human organism can be characterized through volatile organic compounds (VOCs) emitted in breath [8]. Acetone in exhaled breath, as the main volatile component of ketone bodies, represents an attractive alternative to the investigation of blood and urine samples for ketone body detection because of its non-invasive nature [9,10].

Despite attractive prospects, the detection of breath acetone remains challenging. The low concentrations and diverse species of VOCs mandate the use of highly sensitive analytical techniques for reliable quantification. Previous studies have shown a statistically significant relationship between breath acetone and ketones in blood and urine in individuals with diabetes using gas chromatography–mass spectrometry (GC-MS) [2,11]. Although GC-MS can measure the concentration of exhaled acetone, its sample pretreatment, enrichment, and chromatographic separation processes require at least 30 min for the complete analysis of a single sample, resulting in complex and time-consuming procedures that cannot meet rapid detection requirements [12]. Moreover, GC-MS has limited sensitivity and required offline enrichment methods such as solid-phase microextraction (SPME), which may lead to partial VOCs loss [13]. The potential of proton transfer reaction coupled to time-of-flight mass spectrometry (PTR-TOF-MS) for VOCs detection has been shown in applications [14], and has the advantages of high sensitivity, rapid response time, resistance to interference from common atmospheric components, no requirement for sample pretreatment and capability for real-time detection.

Currently, few studies have investigated the correlation of exhaled acetone concentrations and blood/urine ketone body levels in individuals with different glycometabolic status. In this study, we explored this correlation using PTR-TOF-MS in individuals with normoglycemia, prediabetes and diabetes.

## 2. Materials and Methods

### 2.1. Participants

A total of 4000 participants undergoing physical examinations were recruited from Health Management Center of West China Hospital, Sichuan University in Chengdu, China, from February 2024 to April 2025. The participants included people with normoglycemia, prediabetes and diabetes. The classification for glycemic status and diagnostic criteria for prediabetes and diabetes are based on the criteria of 1999 WHO and 2010 ADA [15,16]. Inclusion criteria: all the subjects aged 18–70 years. Patients with diabetes should meet all the following criteria: diabetes duration < 10 years, glycated hemoglobin (HbA1c) < 9.0%, or fasting blood glucose < 11.1 mmol/L. Exclusion criteria: Women in pregnancy and lactation; subjects with ventilation or gas exchange dysfunction, or abnormal liver or kidney functions; chronic alcoholics; smokers; subjects on calorie or carbohydrate restriction diet and spicy food; engaging in vigorous exercise one week before sample collection; diabetic patients receiving insulin therapy; any disability that would affect the use of the devices; and subjects unwilling to complete informed consent.

The patients were assigned into group urine ketone-negative and urine ketone-positive on the basis of results of urinary ketone in the general, normoglycemic, prediabetic and diabetic populations, respectively. Meanwhile, the patients were assigned into 5 groups on the basis of urinary ketone concentration: group urine ketone−, urinary ketone recorded as negative; group urine ketone+, urinary ketone recorded as mild positive; group urine ketone++, urinary ketone recorded as positive; group urine ketone+++, urinary ketone recorded as moderate positive; and group urine ketone++++, urinary ketone recorded as strong positive.

This study conformed to the ethical principles of the Declaration of Helsinki. Meanwhile, the study was approved by an independent ethics committee of the West China Hospital of Sichuan University (Reviewed No. 2488 in 2024). All subjects gave informed consent after a detailed explanation of the study protocol.

### 2.2. Blood and Urine Sample Collection and Analytical Methods

Subjects underwent a physical examination that included weight, height, waist and hip circumference. They kept fasting status from 10 p.m. the preceding evening until the end of the sample (including blood collection breath and urine sample). Blood samples were collected by standard vein puncture into the plain tube for blood glucose and lipid, liver and renal function, and blood βHB. Blood βHB concentrations were measured by the cycling enzyme method (Shanghai Jingyuan Medical Devices Co., Ltd. Shanghai, China). The intra- and inter-assay coefficients of variation were both less than 5%, and the reference range was 0.02–0.27 mmol/L. In this study, a blood βHB concentration > 0.27 mmol/L was used as the cut-off for ketone body positivity based on the aforementioned reference range.

Urinary ketone detection was conducted within one hour after the collection of clean and midstream urine. Urinary ketone was determined by alkaline nitroprusside method Sysmex Wuxi Co., Ltd., Wuxi, China), mainly reacted with AcAc to produce a chromogenic compound. When the concentration of urinary acetone was high, a faint color reaction would occur. According to the urinary ketone detecting package insert, color changes of −, +, ++, +++, and ++++ correspond to concentrations of 0 mmol/L, 0.93 mmol/L, 2.8 mmol/L, 7.4 mmol/L, and higher than 7.4 mmol/L, respectively.

### 2.3. Breath Samples Collection and PTR-TOF MS Analysis

Breath samples were collected using an offline sampling device (PEC.A02, Aliben Science and Technology Co., Ltd., Chengdu, China) equipped with a flow meter and a CO_2_ sensor. This setup allows for the selective capture of endogenous compounds from alveolar gas while minimizing the influence of hyperventilation. The operator inserted a disposable mouthpiece into the handle collection port, and participants breathed into the device in a single, continuous exhalation without pausing or inhaling. The initial dead space gas was diverted through an exhaust line. Once the exhaled CO_2_ concentration reached 4%, the end-tidal alveolar portion was automatically directly into a 500 mL Tedlar bag (500 mL, Dalian Delin Gas Packing Co., Ltd., Dalian, China). The standardized offline sampling system ensures consistent and reliable sample acquisition. All samples were collected between 7:30 and 8:30 a.m. in order of blood, breath and urine sample collection. After collection, the breath sample bags were placed in an insulated container with cooling function (2–5 °C) to prevent volatilization, and transported to the laboratory within one hour. Upon arrival, the samples were kept in the laboratory at 25 °C for analysis. All samples were analyzed within 6 h after collection. Ambient air samples were collected and analyzed in parallel to control for potential background contamination.

Breath acetone analysis was conducted using a PTR-TOF MS 2500 instrument (Aliben Science and Technology Co., Ltd., Chengdu, China). The system consists of a proton transfer reaction (PTR) ion source coupled with an orthogonal acceleration time-of-flight (TOF) mass analyzer. H_3_O^+^ ions are generated from pure water vapor through a cathode discharge and then directed into a drift tube where proton transfer reactions take place. Exhaled VOCs with sufficient proton affinity undergo protonation (VOC + H_3_O^+^ → VOCH^+^ + H_2_O) and are subsequently detected by the TOF analyzer based on their mass-to-charge ratio (*m*/*z*). This state-of-the-art instrument achieves an ultra-low limit of detection (LOD < 10 pptv) and a mass resolution greater than 2500 (m/Δm). Samples were introduced into the PTR-TOF MS via a heated PEEK capillary (inner diameter 2.10 mm, length 1.50 m). To prevent condensation, both the capillary and the ion source were maintained at 70 °C. The drift tube was operated at a pressure of 260 Pa and a reduced electric field (E/N) of 120 Td, where E represents the electric field strength and N is the gas number density. Signal acquisition was carried out using a 1 GS s^−1^ analog-to-digital converter at a sampling frequency of 20 kHz. Each sample was measured for an accumulation time of 50 s, extracting the peak area at *m*/*z* 59. To minimize potential carryover between samples, the sampling pathway was thoroughly flushed with high-purity nitrogen after each measurement, and the next sample was introduced only after the acetone signal at *m*/*z* 59 had returned to a level close to the blank response. The standard acetone gas was diluted to concentrations of 0.5, 1, 2, 5, and 10 ppmv to establish a standard curve of gas concentration versus peak area. The validated calibration range for acetone quantification in this study was 0.5–10 ppmv. The calibration curve showed good linearity over this range: Peak area = 1,161,117.98 × C_acetone + 8972.94, R^2^ = 0.9970. Accordingly, breath acetone concentration was calculated as C_acetone (ppmv) = (peak area − 8972.94)/1,161,117.98. The calibration curve is shown in Supplementary Figure S1.

Precision and accuracy were evaluated using two QC concentrations, 0.5 ppmv and 10 ppmv, representing the lower and upper ends of the validated calibration range. Each QC concentration was measured six times per day over three separate days. The raw peak areas and calculated acetone concentrations are provided in Supplementary Table S1. Intra-day precision was calculated as the relative standard deviation (RSD) of six replicate measurements within the same day. Inter-day precision was calculated as the RSD of all replicate measurements obtained over the three days. The intra-day RSDs were 0.44–0.67% for 0.5 ppmv and 0.48–1.00% for 10 ppmv. The inter-day RSDs were 1.20% and 3.16%, respectively. The estimated limit of detection (LOD) and limit of quantitation (LOQ) were 0.00149 ppmv and 0.00498 ppmv, respectively. These results demonstrated acceptable precision and accuracy for breath acetone quantification within the validated calibration range of 0.5–10 ppmv. The summarized precision and accuracy results are shown in Supplementary Table S2.

### 2.4. Statistical Analysis

All statistical analyses were performed using SPSS statistics software version 25.0 (IBM, Armonk, NY, USA). Qualitative data were expressed as frequency, and chi-square test was used to compare qualitative data between two groups. The mean ± standard deviation (SD) was used to express continuous variables with a normal distribution, and the *t*-test was used for comparison between two groups. The median (25th to 75th percentiles) was used to express continuous variables without a normal distribution. The Mann–Whitney U-test was used for comparison between two groups, and Kruskal–Wallis H-test followed by Dunn‘s post hoc test with Bonferroni correction was used for multiple groups. A Spearman rank correlation analysis was performed to assess the relationship between breath acetone concentration and blood βHB. A receiver operating characteristic (ROC) curve was constructed to determine the optimal cut-off value of concentration of breath acetone for the diagnosis of detecting positive blood βHB, and sensitivity and specificity were calculated. Multiple linear regression was used to analyze the relationship between breath acetone and blood βHB, adjusting for potential confounders. To examine a possible non-linear relationship, a quadratic term of blood βHB was added to the linear model. The improvement in model fit was evaluated by the change in R^2^ (ΔR^2^) and its associated F-test. For the final quadratic model, the inflection point of the curve was calculated as -B_1_/(2B_2_), where B_1_ and B_2_ are the unstandardized regression coefficients for the linear and quadratic terms, respectively. A two-sided test was used, and *p* < 0.05 was considered statistically significant.

## 3. Results

A flow diagram of the study is presented as Supplementary Materials Figure S2. A total of 277 subjects were excluded from the study due to ventilation or gas exchange dysfunction or abnormal liver or kidney function. In total, 3723 cases were included in the final analysis: 2261 with normoglycemia, 1187 with prediabetes and 275 with diabetes.

### 3.1. Clinical Characteristics and Breath Acetone and Blood βHB Concentrations in Group Urine Ketone-Negative and Urine Ketone-Positive

**Figure 1:**
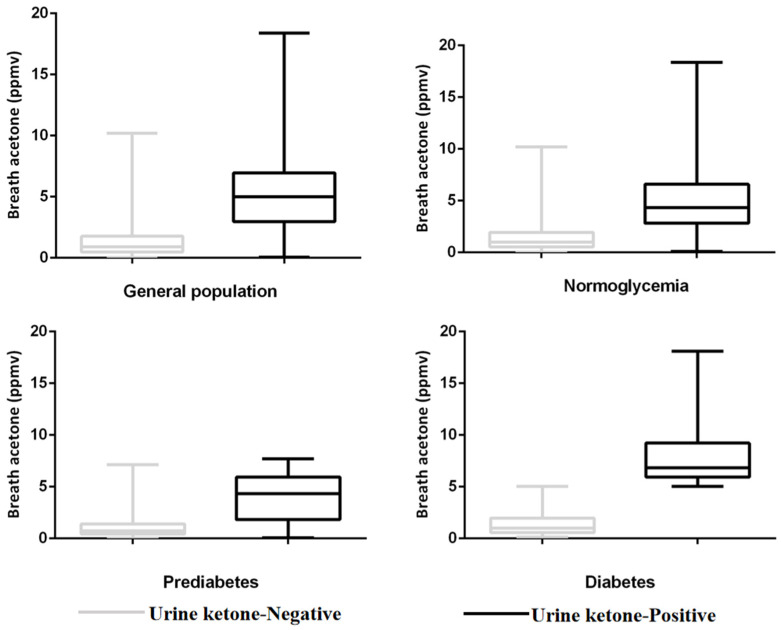
Breath acetone of group urine ketone-negative and urine ketone-positive in individuals with different glycometabolic status.

Blood βHB and breath acetone were significantly higher in group urine ketone-positive than in group urine ketone-negative (*p* < 0.001), indicating that the concentrations of ketone bodies in blood, urine and exhaled breath were relatively consistent (Table 1 and Figure 1). Subjects in group urine ketone-positive were significantly younger than those in group urine ketone-negative (*p* < 0.01) (Table 1 and Supplementary Materials Tables S3–S5). In diabetes, males accounted for a higher proportion in group urine ketone-positive than in group urine ketone-negative (*p* = 0.029) (Supplementary Materials Table S5). In general population and normoglycemia, body mass index (BMI), waist circumference (WC), waist-to-hip ratio, fasting blood glucose (FBG), blood hemoglobin A1c (HbA1c) and triglyceride (TG) were significantly lower in group urine ketone-positive than in group urine ketone-negative (*p* < 0.05). High-density lipoprotein cholesterol (HDL-C) was significantly higher in group urine ketone-positive than in group urine ketone-negative (*p* < 0.001) (Table 1 and Supplementary Materials Table S3). There were no significant differences in BMI, WC, waist-to-hip ratio, FBG, HbA1c, TC, TG, LDL-C, HDL-C, liver or kidney function between two groups in prediabetes and diabetes (*p* > 0.05) (Supplementary Materials Tables S4 and S5).

### 3.2. Comparison of the Concentration of Blood βHB and Breath Acetone in Different Degree of Urine Ketone in General Population

**Figure 2:**
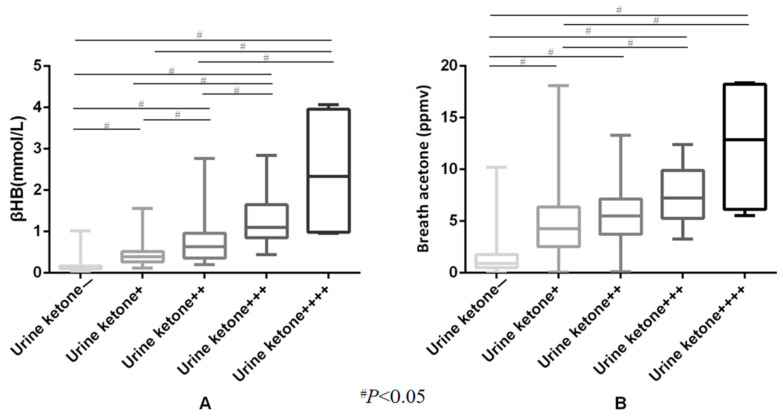
Blood βHB (**A**) and breath acetone (**B**) in different degree of urine ketone in general population.

A progressive elevation in blood βHB and exhaled acetone concentrations was observed with increasing urinary ketone levels (*p* < 0.001) (Table 2 and Figure 2). The concentration of blood βHB was significantly higher in groups urine ketone+++ and urine ketone++++ than in urine ketone++, urine ketone+, and urine ketone− (*p* < 0.05), but there were no differences between groups urine ketone+++ and urine ketone++++. The concentration of exhaled acetone was significantly higher in groups urine ketone+++ and urine ketone++++ than in urine ketone+ and urine ketone− (*p* < 0.05), but there were no differences among groups urine ketone++, urine ketone+++, and urine ketone++++.

### 3.3. The Spearman Rank Correlation Between Breath Acetone and Blood βHB in Different Glycometabolic Status

**Figure 3:**
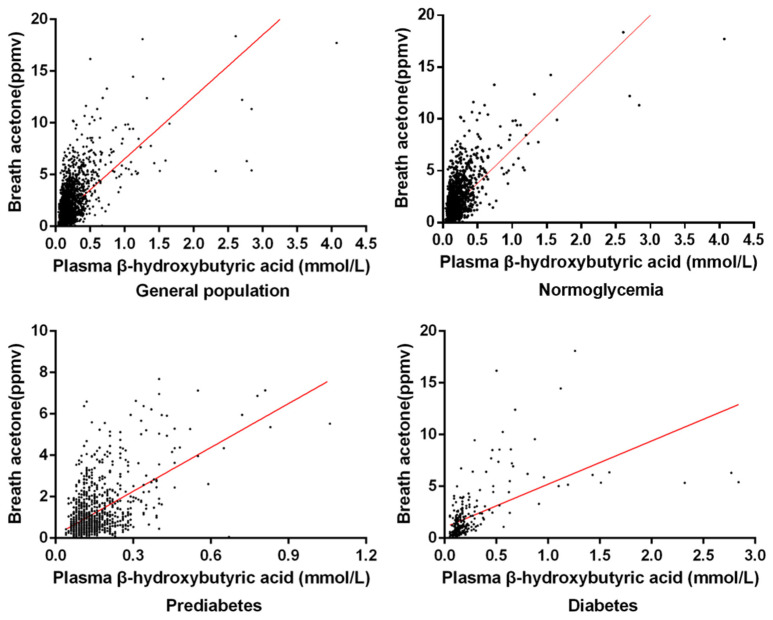
Correlation analysis of blood βHB and breath acetone in different glycometabolic status.

The Spearman rank correlation coefficient of breath acetone and blood βHB was 0.493, 0.522, 0.379, and 0.636 in general population, normoglycemia, prediabetes, and diabetes, respectively. The concentration of breath acetone was positively correlated with the concentration of blood βHB, with strongest correlation in diabetes and weakest correlation in prediabetes (Table 3 and Figure 3).

### 3.4. Diagnostic Value and Preliminary Thresholds of Breath Acetone Concentration for Detecting Blood βHB Positivity

**Figure 4:**
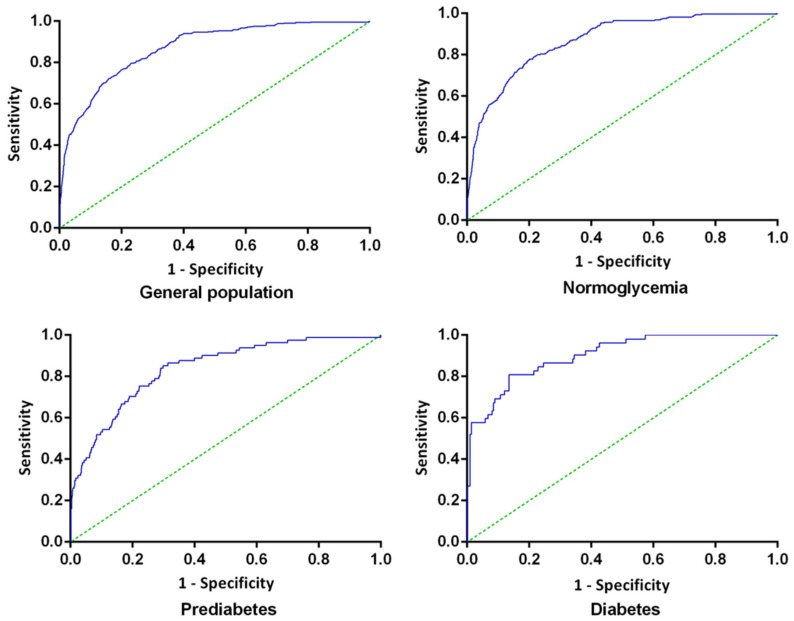
Receiver operating characteristic (ROC) curve for breath acetone in detecting blood βHB positivity.

With a positive blood βHB result (>0.27 mmol/L) defined as the diagnostic criterion, ROC curve analysis was used to evaluate the preliminary thresholds of breath acetone for detecting blood βHB positivity (Figure 4). The area under the ROC curve (AUC) of breath acetone was highest in diabetes and the cut-off value was 2.37 ppmv, which was obviously higher than that in normoglycemia and prediabetes, with a sensitivity of 80.8% and a specificity of 86.5%. The cut-off values were 1.10 ppmv in prediabetes, with a sensitivity of 85.2% and a specificity of 70.2%, and 2.05 ppmv in normoglycemia, with a sensitivity of 77.9% and a specificity of 79.9% (Table 4).

### 3.5. Multiple Linear Regression and Non-Linear Relationship Between Breath Acetone and Blood βHB

The primary predictor, blood βHB, was initially entered into a linear model together with the following covariates selected a priori: age, BMI, WC, waist-to-hip ratio, FBG, HbA1c, TG and HDL-C. Because of strong collinearity among the adiposity indices (WC and waist-to-hip ratio showed a variance inflation factor (VIF) > 10; only BMI was retained in the final adjusted models.

**Figure 5:**
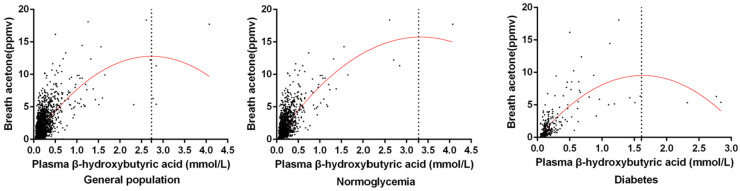
The dose–response curve between breath acetone and blood βHB.

Multiple linear regression with a quadratic term for blood βHB was performed to examine the relationship between breath acetone and blood βHB after adjusting for age, BMI, FBG, HbA1c, TG and HDL-C. In general population, the overall model was statistically significant (F_(8,3705)_ = 395.66, *p* < 0.001), explaining 46.1% of the variance in breath acetone concentration (R^2^ = 0.461, adjusted R^2^ = 0.460). As shown in Table 5, blood βHB exhibited a significant quadratic association with breath acetone. The linear term was positive and significant (B = 9.269, *p* < 0.001), while the quadratic term was negative and significant (B = −1.698, *p* < 0.001), indicating an inverted U-shaped (concave) relationship. The inflection point of the quadratic curve was calculated as −B_1_/(2B_2_) = −9.269/(2 × −1.698) ≈ 2.73 mmol/L of blood βHB, beyond which breath acetone decreased with increasing blood βHB (Figure 5). In normoglycemia and diabetes, breath acetone also exhibited an inverted U-shaped relationship with blood βHB (B = −1.409/−16.070, *p* < 0.001) (Supplementary Materials Tables S6 and S8). The inflection points of the quadratic curve were 3.28 mmol/L and 1.61 mmol/L of blood βHB, respectively (Figure 5). In prediabetes, the linear term of blood βHB was positively and significantly associated with breath acetone (B = 7.582, *p* < 0.001), and the quadratic term (βHB^2^) was not statistically significant (B = −0.949, *p* = 0.412) (Supplementary Materials Table S7).

## 4. Discussion

This study confirmed that breath acetone concentration using PTR-TOF-MS was associated with ketone bodies in blood and urine in people with normoglycemia, prediabetes and diabetes. A progressive elevation in breath acetone concentrations was observed with increasing urinary ketone levels. In normoglycemia and diabetes, breath acetone exhibited an inverted U-shaped relationship with blood βHB, while in prediabetes, breath acetone showed a linear positive correlation with blood βHB.

Compared with conventional methods such as GC-MS, PTR-TOF-MS requires no complex sample pretreatment and, as a powerful tool for real-time detection of VOCs in exhaled breath, enhances the temporal resolution, precision, and efficiency of analysis [17,18,19]. This study measured breath acetone concentrations using PTR-TOF-MS. Previous studies investigated the relationship between breath acetone and blood and urine ketones in patients with type 1 and type 2 diabetes mellitus [11,20], but few have focused on people with normoglycemia and prediabetes. While diabetic ketoacidosis is a critical emergency, mild ketosis offers a contrasting benefit. It promotes resistance to oxidative and inflammatory stress, improving metabolism, extending lifespan, and enhancing neurological responses [21,22]. Therefore, investigating the relationship between breath acetone and blood and urine ketones is important not only for people with diabetes but also for those with normoglycemia and prediabetes. Our findings demonstrated that blood βHB and breath acetone were significantly higher in the urine ketone-positive group than in the urine ketone-negative group in people with different glycemic statuses, indicating consistency among ketone body measurements in urine, blood, and exhaled breath in people with normoglycemia, prediabetes, and diabetes. This consistency supports the potential utility of breath acetone analysis using PTR-TOF-MS as a non-invasive alternative for monitoring ketone levels, which could be particularly valuable for patients requiring frequent ketone monitoring, such as those with type 1 diabetes or individuals following ketogenic dietary interventions [23].

Studies on diabetes have shown that younger patients were more likely to experience ketosis [2,24]. In this study, regardless of whether they had diabetes, prediabetes, or normoglycemia, participants with positive ketone bodies were also younger. This age-related difference might be attributed to higher metabolic flexibility and greater capacity for fatty acid mobilization in younger individuals. Our results showed that in individuals with diabetes, the proportion of males was higher in the urine ketone-positive group. Clinical evidence has shown that male patients with diabetes, particularly those with ketosis-prone type 2 diabetes, have a higher risk of developing ketosis compared to females. A review by Wang and Tan (2015) reported a two- to threefold higher prevalence of ketosis-prone diabetes mellitus (KPDM) in men, with male-to-female ratios ranging from 1.5:1 to 6:1 [25]. The underlying mechanisms may involve gender-related body fat distribution, hormonal and genetic factors, which are associated with the diabetic process and glucose homeostasis and metabolism. An early study on clinical diabetes showed that the higher the FBG, the higher the urinary ketone concentration [2]. This study found no significant differences in FBG and HbA1c between the urine ketone-positive group and the urine ketone-negative group in individuals with prediabetes and diabetes. This might be related to the increase in euglycemic diabetic ketosis that occurred due to the recent use of SGLT-2 inhibitors [26,27]. Generally, blood glucose and ketone bodies vary between individuals depending on diet, medications, stress, and physical activities [11,28,29]. In the general population of our study, BMI, WC, waist-to-hip ratio, FBG, HbA1c, and TG were significantly lower, and HDL-C was significantly higher, in the urine ketone-positive group than in the urine ketone-negative group, indicating that lipid mobilization was more likely to occur during fasting in individuals with a lean body type and better glucose and lipid metabolism, mainly because increased ketogenesis resulting from lipolysis is more likely to occur in the context of low insulin levels and good insulin sensitivity [22,30].

Our correlation analysis showed a weak positive correlation between breath acetone and blood βHB. The Spearman rank correlation coefficient was 0.493, 0.522, 0.379, and 0.636 in general population, normoglycemia, prediabetes, and diabetes, respectively. Saasa V et al. and Qiao Y et al. reported correlation coefficients of r = 0.821 and 0.817, respectively, between breath acetone and blood βHB individuals with diabetes [2,11], indicating a strong correlation. However, other investigations found strong correlations only at low acetone/βHB levels, with the correlation weakening at higher levels [31]. Blood–breath data from multiple studies have demonstrated a strong non-linear relationship between breath acetone and blood βHB [29]. Our multiple linear regression showed that breath acetone exhibited an inverted U-shaped relationship with blood βHB in normoglycemia and diabetes; the inflection points of the quadratic curve were 3.28 mmol/L and 1.61 mmol/L of blood βHB, respectively. Acetone is generated by the spontaneous decarboxylation of AcAc, while βHB and AcAc are interconverted by β-hydroxybutyrate dehydrogenase in a reaction tightly coupled to the mitochondrial NAD^+^/NADH redox state. As ketosis intensifies—particularly in insulin-deficient states—the hepatic NADH/NAD^+^ ratio rises, shifting the equilibrium markedly towards βHB. Consequently, the AcAc pool available for acetone production becomes depleted, causing breath acetone to decline even as blood βHB continues to rise [1]. This redox shift explains both the descending limb of the inverted U-shaped curve and the observation that the inflection point occurs at a substantially lower βHB concentration in diabetes (1.61 mmol/L) than in normoglycemia (3.28 mmol/L). In diabetes, chronic insulin deficiency or resistance may provoke an earlier and more pronounced rise in the NADH/NAD^+^ ratio, thereby suppressing acetoacetate and breath acetone at a lower βHB threshold. The quadratic term was not significant in prediabetes, yielding only a positive linear association. As shown in Supplementary Tables S4 and S5, breath acetone was lower in prediabetes than in diabetes. This suggests that, over the range of βHB observed in our prediabetic participants, the relationship has not yet entered its descending phase.

The present study investigated the preliminary thresholds of breath acetone for detecting blood βHB positivity. The AUC was highest in diabetes, with a cut-off value of 2.37 ppmv yielding a sensitivity of 80.8% and a specificity of 86.5%. The higher threshold compared with those in normoglycemia and prediabetes likely reflects the insulin deficiency of diabetic patients. Increased lipolysis occurs under conditions of low insulin levels [22]. Tsunemi et al. reported an optimal breath acetone cut-off of 3400 ppb (approximately 3.4 ppmv) for predicting diabetic ketoacidosis risk. The lower cut-off value in our study was likely on account of the difference in reference standard (blood βHB > 0.27 mmol/L versus blood total ketone bodies ≥ 1000 µmol/L) [32]. In normoglycemic individuals, mild ketosis may occur during brief fasting [29]. Our results showed that the optimal cut-off values were 2.05 ppmv for normoglycemia (sensitivity 77.9%, specificity 79.9%) and 1.10 ppmv for prediabetes (sensitivity 85.2%, specificity 70.2%). While these results indicate that breath acetone can discriminate between individuals with and without elevated βHB, the interpretation of these ROC-derived thresholds must be placed in the context of our finding of an inverted U-shaped relationship between breath acetone and blood βHB in normoglycemia and diabetes. The ROC analysis, which relies on a binary outcome anchored at a low threshold of >0.27 mmol/L, is inherently weighted towards the rising portion of the inverted U-shaped curve and cannot account for this diagnostic blind spot in the higher βHB range. In prediabetes, breath acetone and blood βHB exhibited a purely positive linear relationship, and the ROC cut-off value could be used to estimate blood βHB levels. These thresholds require further validation in independent, external cohorts before they can be considered for clinical application.

In view of the invasiveness of blood βHB testing and the time lag of urine ketone measurement, it is important to develop breath-acetone-based approaches for non-invasive ketone monitoring. The study investigated the correlation between breath acetone and ketone bodies in blood and urine. This study only explored the relationship in the fasting state, and we did not collect detailed information on participants’ dietary patterns, physical activity levels, or medication use, all of which could influence ketone body concentrations. In further studies, the relationship among them in different stages of DKA and relative to the status of vigorous exercise and calorie or carbohydrate restriction diet should also be discussed. Meanwhile, the present study did not compare PTR-TOF-MS with other acetone detection methods (e.g., GC-MS, semiconductor gas sensors). Our future studies will systematically evaluate the analytical and clinical performance of different methods, including their advantages and limitations, to further validate the clinical applicability of PTR-TOF-MS.

## 5. Conclusions

The PTR-TOF-MS was used to successfully quantify the concentration of breath acetone in people with normoglycemia, prediabetes and diabetes. A progressive elevation in breath acetone concentrations was observed with increasing urinary ketone levels. Breath acetone showed a glycemic-status-dependent relationship with blood βHB. While the overall correlation was moderate, the association followed an inverted U-shaped curve in normoglycemic and diabetic individuals, but remained linear in prediabetes. These findings support the potential of breath acetone as a non-invasive ketone monitoring tool, but highlight that its clinical interpretation must account for glycemic status and the level of blood βHB.

## Figures and Tables

**Table 1:** Clinical characteristics and ketone body concentration of group urine ketone-negative and urine ketone-positive in general population.

Variables	Urine Ketone-Negative (n = 3525)	Urine Ketone-Positive (n = 198)	*p*-Value
Age (yr)	48.05 ± 10.88	40.96 ± 10.81	<0.001
Sex (male, n, (%))	1260 (35.74)	78 (39.39)	0.761
BMI (kg/m^2^)	23.29 ± 3.18	21.95 ± 3.17	<0.001
WC (cm)	77.80 ± 9.83	73.59 ± 10.07	<0.001
Waist-to-hip ratio	0.83 ± 0.07	0.80 ± 0.08	<0.001
FBG (mmol/L)	4.79 (4.49, 5.16)	4.54 (4.17, 4.92)	<0.001
HbA1c (%)	5.60 (5.30, 5.80)	5.40 (5.20, 5.70)	<0.001
TC (mmol/L)	4.85 ± 0.89	4.79 ± 0.96	0.366
TG (mmol/L)	1.43 ± 1.51	1.15 ± 1.46	0.011
LDL-C (mmol/L)	2.87 (2.38, 3.39)	2.78 (2.24, 3.40)	0.096
HDL-C (mmol/L)	1.39 (1.17, 1.68)	1.56 (1.25, 1.85)	<0.001
ALT (U/L)	17.00 (13.00, 23.00)	14.00 (10.25, 20.00)	0.064
AST (U/L)	20.00 (17.00, 23.00)	18.50 (17.00, 23.00)	0.075
BUN (mmol/L)	4.90 (4.10, 5.70)	4.80 (4.00, 6.00)	0.545
Cr (μmol/L)	67.90 ± 11.51	67.14 ± 12.79	0.369
Blood βHB (mmol/L)	0.12 (0.10, 0.17)	0.44 (0.30, 0.67)	<0.001
Breath acetone (ppmv)	0.91 (0.49, 1.77)	5.00 (2.98, 6.96)	<0.001

BMI, body mass index; WC, waist circumference; FBG, fasting blood glucose; HbA1c, blood hemoglobin A1c; TC, total cholesterol; TG, triglyceride; LDL-C, low-density lipoprotein cholesterol; HDL-C, high-density lipoprotein cholesterol; ALT, alanine transaminase; AST, aspartate aminotransferase; BUN, blood urea nitrogen; Cr, creatinine; βHB, blood β-hydroxybutyrate. Note: All values are expressed as mean (standard deviation), number/proportion or median (25th to 75th percentiles).

**Table 2:** Comparison of the concentration of blood βHB and breath acetone in different degree of urine ketone.

Variables	Urine Ketone− (n = 3525)	Urine Ketone+ (n = 132)	Urine Ketone++ (n = 43)	Urine Ketone+++ (n = 19)	Urine Ketone++++ (n = 4)	*p*-Value
Blood βHB (mmol/L)	0.12 (0.10, 0.17)	0.39 (0.27, 0.52)	0.63 (0.36, 0.96)	1.10 (0.85, 1.65)	1.84 (0.99, 3.71)	<0.001
Breath acetone (ppmv)	0.91 (0.49, 1.77)	4.27 (2.52, 6.36)	5.51 (3.73, 7.13)	7.24 (5.27, 9.91)	12.87 (6.15, 18.22)	<0.001

βHB, blood β-hydroxybutyrate. Note: All values are expressed as median.

**Table 3:** The Spearman rank correlation coefficient of breath acetone and blood βHB in different glycometabolic status.

	ρ	*p*-Value
General population	0.493	<0.001
Normoglycemia	0.522	<0.001
Prediabetes	0.379	<0.001
Diabetes	0.636	<0.001

**Table 4:** Discriminatory accuracy and preliminary thresholds of breath acetone for detecting blood βHB positivity.

	Sensitivity	Specificity	AUC (95%CI)	*p*-Value	Youden Index	Cut-Off
General population	0.765	0.802	0.871 (0.853–0.889)	<0.001	0.568	1.89
Normoglycemia	0.779	0.799	0.870 (0.849–0.891)	<0.001	0.578	2.05
Prediabetes	0.852	0.702	0.838 (0.793–0.884)	<0.001	0.553	1.10
Diabetes	0.808	0.865	0.902 (0.858–0.946)	<0.001	0.673	2.37

**Table 5:** Regression coefficients for variables predicting breath acetone in general population.

Variables	Unstandardized B (SE)	Standardized β	t	*p*-Value	Tolerance	VIF
(Constant)	0.783 (0.297)		2.642	0.008		
Blood βHB (linear)	9.269 (0.216)	0.975	42.937	<0.001	0.282	3.54
Blood βHB^2^ (quadratic)	−1.698 (0.092)	−0.416	−18.504	<0.001	0.288	3.47
Age	−0.012 (0.002)	−0.077	−5.879	<0.001	0.851	1.175
BMI	0.015 (0.008)	0.028	2.009	0.045	0.735	1.361
HbA1c	−0.182 (0.057)	−0.068	−3.199	0.001	0.325	3.076
FBG	0.096 (0.034)	0.059	2.878	0.004	0.344	2.903
TG	−0.030 (0.015)	−0.026	−2.008	0.045	0.842	1.188
HDL-C	0.078 (0.066)	0.017	1.189	0.235	0.698	1.432

BMI, body mass index; HbA1c, blood hemoglobin A1c; FBG, fasting blood glucose; TG, triglyceride; HDL-C, high-density lipoprotein cholesterol; VIF, variance inflation factor.

## Data Availability

The data analyzed in this study is subject to the following licenses/restrictions: The raw data supporting the conclusions of this article will be made available by the authors, without undue reservation. Requests to access these datasets should be directed to Y.H., yanhuang@wchscu.edu.cn.

## Supplementary Materials

The following supporting information can be downloaded at: https://www.mdpi.com/article/10.3390/diagnostics16132043/s1. Figure S1. Calibration curve of acetone by PTR-TOF-MS at m/z 59. Figure S2. A flow diagram for the study. Table S1. Peak areas and calculated concentrations of acetone QC samples for precision assessment. Table S2. Precision and accuracy of acetone quantification by PTR-TOF-MS. Table S3. Clinical characteristics and ketone body concentration of group urine ketone-negative and urine ketone-positive in normoglycemia. Table S4. Clinical characteristics and ketone body concentration of group urine ketone-negative and urine ketone-positive in prediabetes. Table S5. Clinical characteristics and ketone body concentration of group urine ketone-negative and urine ketone-positive in diabetes. Table S6. Regression coefficients for variables predicting breath acetone in normoglycemia. Table S7. Regression coefficients for variables predicting breath acetone in prediabetes. Table S8. Regression coefficients for variables predicting breath acetone in diabetes.

## Abbreviations

The following abbreviations are used in this manuscript:

PTR-TOF-MS	Proton transfer reaction coupled to time-of-flight mass spectrometry
βHB	Β-hydroxybutyrate
ROC	Receiver operating characteristic
VOCs	Volatile organic compounds
GC-MS	Gas chromatography–mass spectrometry
RSD	Relative standard deviation
LOD	Limit of detection
LOQ	Limit of quantitation
DKA	Diabetic ketoacidosis
SGLT-2	Sodium-glucose transporter type 2
BMI	Body mass index
WC	Waist circumference
FBG	Fasting blood glucose
HbA1c	Blood hemoglobin a1c
TG	Triglyceride
HDL-C	High-density lipoprotein cholesterol
