# Supplementary material for: Correlation Between Breath Acetone and Ketone Bodies in Blood and Urine Among Individuals with Different Glycometabolic Statuses Based on PTR-TOF-MS
Source: Diagnostics (Basel). 2026 Jun 30;16(13):2043. doi: 10.3390/diagnostics16132043 (PMC13360147; doi:10.3390/diagnostics16132043)
Supplement: Supplementary file 1 [file diagnostics-16-02043-s001.zip › diagnostics-4319372-supplementary.pdf]

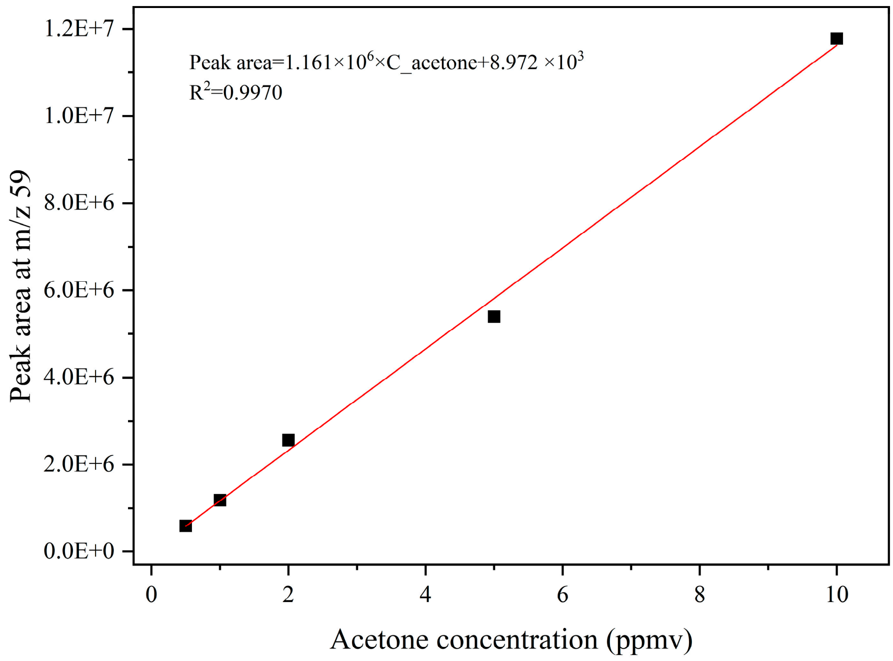

Figure S1 Calibration curve of acetone by PTR-TOF-MS at *m/z* 59

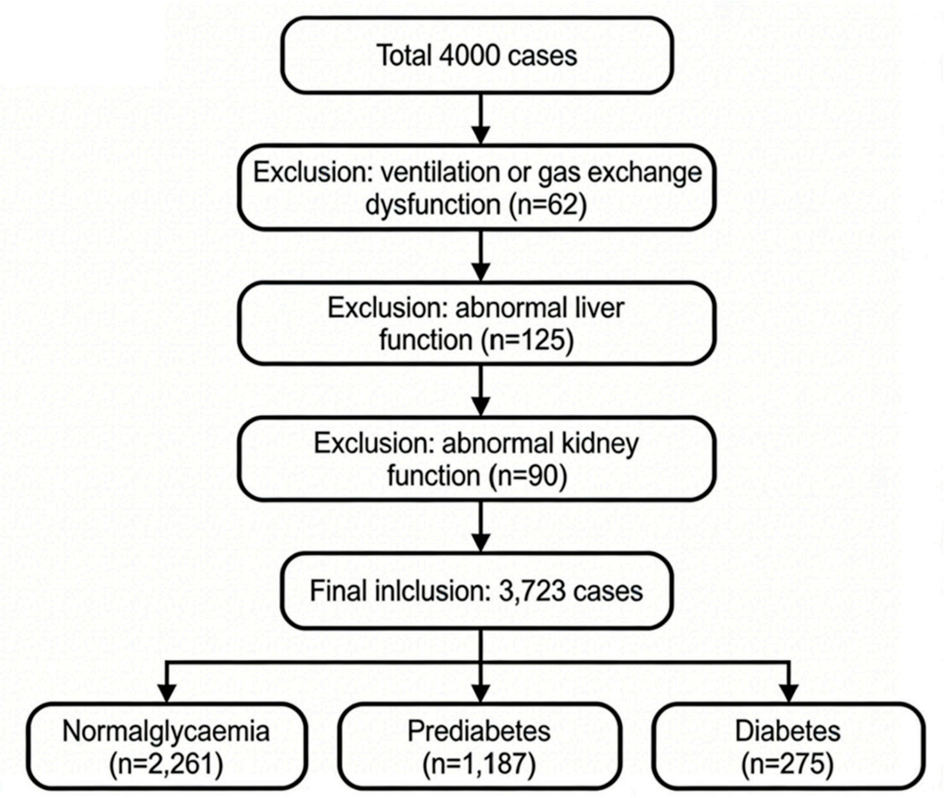

Figure S2. A flow diagram for the study

Table S1. Peak areas and calculated concentrations of acetone QC samples for precision assessment

Day	Nominal concentration (ppmv)	Replicate	Peak area at m/z 59	Calculated concentration (ppmv)	Nominal concentration (ppmv)	Replicate	Peak area at *m/z* 59	Calculated concentration (ppmv)
1	0.5	1	594345.9020	0.5041	10	1	11596481.7143	9.9796
1	0.5	2	601706.8889	0.5105	10	2	11795553.9429	10.1511
1	0.5	3	600144.0147	0.5091	10	3	11855521.3767	10.2027
1	0.5	4	599348.8000	0.5085	10	4	11820981.1000	10.1730
1	0.5	5	600787.1538	0.5097	10	5	11629876.3179	10.0084
1	0.5	6	598748.0755	0.5079	10	6	11763509.6409	10.1235
2	0.5	1	589020.8222	0.4996	10	1	11902278.7660	10.2430
2	0.5	2	584448.5593	0.4956	10	2	12124899.1764	10.4347
2	0.5	3	586339.7143	0.4973	10	3	11964402.8814	10.2965

2	0.5	4	588592.1064	0.4992	10	4	11966234.6560	10.2981
2	0.5	5	579733.0588	0.4916	10	5	11754934.5091	10.1161
2	0.5	6	582148.3519	0.4936	10	6	11964718.3454	10.2968
3	0.5	1	590441.9831	0.5008	10	1	12546014.5231	10.7974
3	0.5	2	581426.2241	0.4930	10	2	12543393.0833	10.7951
3	0.5	3	586021.2000	0.4970	10	3	12615359.7000	10.8571
3	0.5	4	590597.6604	0.5009	10	4	12678025.5000	10.9111
3	0.5	5	590546.1837	0.5009	10	5	12519916.6779	10.7749
3	0.5	6	591009.2941	0.5013	10	6	12621734.6560	10.8626

Table S2. Precision and accuracy of acetone quantification by PTR-TOF-MS

QC level	Nominal concentration (ppmv)	Intra-day RSD range (%)	Inter-day measured concentration, mean ± SD (ppmv)	Inter-day RSD (%)
Low	0.5	0.44-0.67	0.5011±0.0060	1.20
High	10	0.48-1.00	10.4067±0.3286	3.16

Table S3. Clinical characteristics and ketone body concentration of group urine ketone-negative and urine ketone-positive in normoglycemia.

Variables	Urine ketone-negative（n=2114）	Urine ketone-positive（n=147）	*p*-Value
Age (yr)	44.49±10.50	37.92±9.41	< 0.001
Sex [Male, n, (%)]	658 (31.13)	40 (27.21)	0.098
BMI (kg/m2)	22.64±3.00	21.35±2.98	< 0.001
WC(cm)	75.59±9.21	70.88±8.70	< 0.001
Waist-to-hip ratio	0.81±0.07	0.77±0.06	< 0.001
FBG (mmol/L)	4.66±0.40	4.37±0.44	< 0.001
HbA1c (%)	5.35±0.22	5.28±0.23	< 0.001
TC (mmol/L)	4.75±0.85	4.78±0.93	0.612
TG (mmol/L)	1.01 (0.74, 1.45)	0.74 (0.58, 1.02)	< 0.001
LDL-C (mmol/L)	2.81±0.72	2.80±0.80	0.864
HDL-C (mmol/L)	1.49±0.38	1.65±0.43	< 0.001
ALT (U/L)	17.08±7.00	16.51±7.76	0.065
AST (U/L)	19.70±4.54	19.26±5.14	0.257
BUN (mmol/L)	4.86±1.24	4.82±1.25	0.737
Cr (μmol/L)	67.07±11.33	66.47±11.58	0.065
Blood βHB (mmol/L)	0.12 (0.10, 0.17)	0.41 (0.30, 0.63)	< 0.001
Breath acetone (ppmv)	1.01 (0.53, 1.94)	4.33 (2.83, 6.59)	< 0.001

BMI, body mass index; WC, waist circumference; FBG, fasting blood-glucose; HbA1c, blood hemoglobin A1c; TC, total cholesterol; TG, triglyceride; LDL-C, low density lipoprotein cholesterol; HDL-C, high density lipoprotein cholesterol; ALT, alanine transaminase; AST, aspartate aminotransferase; BUN, blood urea nitrogen; Cr, creatinine; βHB, blood β-hydroxybutyrate. Note: All values are expressed as

mean (standard deviation), number/proportion or median (25th to 75th percentiles).

Table S4. Clinical characteristics and ketone body concentration of group urine ketone-negative and urine ketone-positive in prediabetes.

**Variables**	**Urine ketone-negative（n=1164）**	**Urine ketone-positive （n=23）**	***p*-Value**
Age (yr)	52.84±9.24	47.74±9.03	0.009
Sex [Male, n, (%)]	430 (36.94)	13 (56.52)	0.055
BMI (kg/m2)	24.07±3.19	23.24±3.63	0.218
WC(cm)	80.13±9.70	78.09±10.62	0.318
Waist-to-hip ratio	0.85±0.07	0.84±0.07	0.459
FBG (mmol/L)	5.05±0.53	5.08±0.60	0.765
HbA1c (%)	5.89±0.18	5.89±0.20	0.935
TC (mmol/L)	5.06±0.88	4.89±0.77	0.353
TG (mmol/L)	1.59±2.00	1.39±1.16	0.633
LDL-C (mmol/L)	3.08±0.77	2.99±0.78	0.930
HDL-C (mmol/L)	1.36 (1.15, 1.61)	1.24 (1.02, 1.73)	0.493
ALT (U/L)	18.00 (14.00, 24.00)	18.00 (12.00, 32.00)	0.686
AST (U/L)	21.00 (18.00, 24.00)	20.00 (17.00, 28.00)	0.663
BUN (mmol/L)	5.10 (4.30, 5.90)	5.30 (4.60, 6.70)	0.178
Cr (*μ*mol/L)	68.00 (60.00, 78.00)	73.00 (60.00, 89.00)	0.082
Blood βHB (mmol/L)	0.12 (0.10, 0.16)	0.45 (0.27, 0.62)	< 0.001
Breath acetone (ppmv)	0.74 (0.43, 1.40)	4.35 (1.82, 5.94)	< 0.001

BMI, body mass index; WC, waist circumference; FBG, fasting blood-glucose; HbA1c, blood hemoglobin A1c; TC, total cholesterol; TG, triglyceride; LDL-C, low density lipoprotein cholesterol; HDL-C, high density lipoprotein cholesterol; ALT, alanine transaminase; AST, aspartate aminotransferase; BUN, blood urea nitrogen; Cr, creatinine; βHB, blood β-hydroxybutyrate. Note: All values are expressed as mean (standard deviation), number/proportion or median (25th to 75th percentiles).

Table S5. Clinical characteristics and ketone body concentration of group urine ketone-negative and urine ketone-positive in diabetes.

Variables	Urine ketone-negative（n=247）	Urine ketone-positive（n=28）	*p*-Value
Age (yr)	56.00±8.00	51.36±10.32	0.005
Sex [Male, n, (%)]	172 (69.64)	25 (89.29)	0.029
BMI (kg/m2)	25.09±3.10	24.00±2.62	0.077
WC(cm)	85.74±8.89	84.11±8.17	0.353
Waist-to-hip ratio	0.90±0.06	0.90±0.07	0.797
FBG (mmol/L)	6.85 (5.95, 7.83)	6.75 (6.06, 9.54)	0.399
HbA1c (%)	6.80 (6.50, 7.50)	7.2 (6.60, 7.90)	0.090
TC (mmol/L)	4.74±1.05	4.75±1.26	0.978
TG (mmol/L)	1.67 (1.10, 2.33)	1.19 (0.92, 2.51)	0.086
LDL-C (mmol/L)	2.81±0.89	2.62±1.12	0.291
HDL-C (mmol/L)	1.18 (0.99, 1.38)	1.18 (1.00, 1.57)	0.280
ALT (U/L)	21.00 (16.00, 28.00)	17.00 (14.00, 37.00)	0.266
AST (U/L)	21.00 (17.00, 24.00)	21.00 (18.00, 31.00)	0.204
BUN (mmol/L)	5.92±1.31	6.20±1.55	0.131
Cr (*μ*mol/L)	72.24±12.86	75.64±11.56	0.181
Blood βHB (mmol/L)	0.13 (0.10, 0.18)	0.66 (0.50, 1.26)	< 0.001
Breath acetone (ppmv)	1.01 (0.55, 1.96)	6.83 (5.94, 9.23)	< 0.001

BMI, body mass index; WC, waist circumference; FBG, fasting blood-glucose; HbA1c, blood hemoglobin A1c; TC, total cholesterol; TG, triglyceride; LDL-C, low density lipoprotein cholesterol; HDL-C, high density lipoprotein cholesterol; ALT, alanine transaminase; AST, aspartate aminotransferase; BUN, blood urea nitrogen; Cr, creatinine; βHB, blood β-hydroxybutyrate. Note: All values are expressed as mean (standard deviation), number/proportion or median (25th to 75th percentiles).

Table S6. Regression coefficients for variables predicting breath acetone in normoglycemia.

Variables	Unstandardized B (SE)	Standardized β	t	*p*-Value	Tolerance	VIF
(Constant)	3.557 (0.717)		4.96	<0.001		
Blood βHB (linear)	9.232 (0.267)	0.955	34.543	<0.001	0.291	3.437
Blood βHB^2 (quadratic)	-1.409 (0.106)	-0.361	-13.300	<0.001	0.302	3.31
Age	-0.009 (0.003)	-0.052	-3.207	0.001	0.851	1.174
BMI	0.029 (0.011)	0.048	2.714	0.007	0.716	1.397
HbA1c	-0.564 (0.130)	-0.069	-4.336	<0.001	0.879	1.138

FBG	-0.129 (0.072)	-0.029	-1.793	0.073	0.829	1.206
TG	-0.030 (0.027)	-0.019	-1.115	0.265	0.799	1.251
HDL-C	0.012 (0.086)	0.003	0.142	0.887	0.673	1.485

BMI, body mass index; HbA1c, blood hemoglobin A1c; FBG, fasting blood-glucose; TG, triglyceride;

HDL-C, high density lipoprotein cholesterol; VIF, variance inflation factor

Table S7. Regression coefficients for variables predicting breath acetone in prediabetes.

**Variables**	**Unstandardized B (SE)**	**Standardized β**	**t**	***p*-Value**	**Tolerance**	**VIF**
(Constant)	1.733 (0.969)		1.788	0.074		
Blood βHB (linear)	7.582 (0.790)	0.577	9.603	<0.001	0.166	6.032
Blood $βHB^2$ (quadratic)	-0.949 (1.156)	-0.049	-0.821	0.412	0.169	5.914
Age	-0.003 (0.003)	-0.020	-0.795	0.427	0.924	1.083
BMI	0.005 (0.010)	0.014	0.504	0.614	0.775	1.29
HbA1c	-0.357 (0.168)	-0.056	-2.119	0.034	0.867	1.153
FBG	0.087 (0.059)	0.039	1.464	0.144	0.847	1.181
TG	-0.011 (0.015)	-0.019	-0.746	0.456	0.895	1.117
HDL-C	0.040 (0.094)	0.012	0.422	0.673	0.712	1.405

BMI, body mass index; HbA1c, blood hemoglobin A1c; FBG, fasting blood-glucose; TG, triglyceride;

HDL-C, high density lipoprotein cholesterol; VIF, variance inflation factor

Table S8. Regression coefficients for variables predicting breath acetone in diabetes.

**Variables**	**Unstandardized B (SE)**	**Standardized β**	**t**	***p*-Value**	**Tolerance**	**VIF**
(Constant)	-6.248 (6.107)		-1.023	0.307		
Blood βHB (linear)	51.700 (3.475)	1.645	14.877	<0.001	0.138	7.231
Blood $βHB^2$ (quadratic)	-16.070 (1.512)	-1.186	-10.629	<0.001	0.136	7.363
Age	-0.053 (0.054)	-0.041	-0.974	0.331	0.95	1.052
BMI	0.028 (0.147)	0.008	0.191	0.849	0.948	1.055
HbA1c	0.060 (0.589)	0.007	0.102	0.919	0.353	2.832
FBG	0.097 (0.310)	0.022	0.314	0.754	0.341	2.929
TG	0.357 (0.295)	0.058	1.211	0.227	0.736	1.358
HDL-C	4.824 (1.515)	0.146	3.184	0.002	0.804	1.244

BMI, body mass index; HbA1c, blood hemoglobin A1c; FBG, fasting blood-glucose; TG, triglyceride;

HDL-C, high density lipoprotein cholesterol; VIF, variance inflation factor
